# Supplementary figures and images for: CellNetVis: a web tool for visualization of biological networks using force-directed layout constrained by cellular components
Source: BMC Bioinformatics. 2017 Sep 13;18(Suppl 10):395. doi: 10.1186/s12859-017-1787-5 (PMC5606216; doi:10.1186/s12859-017-1787-5)

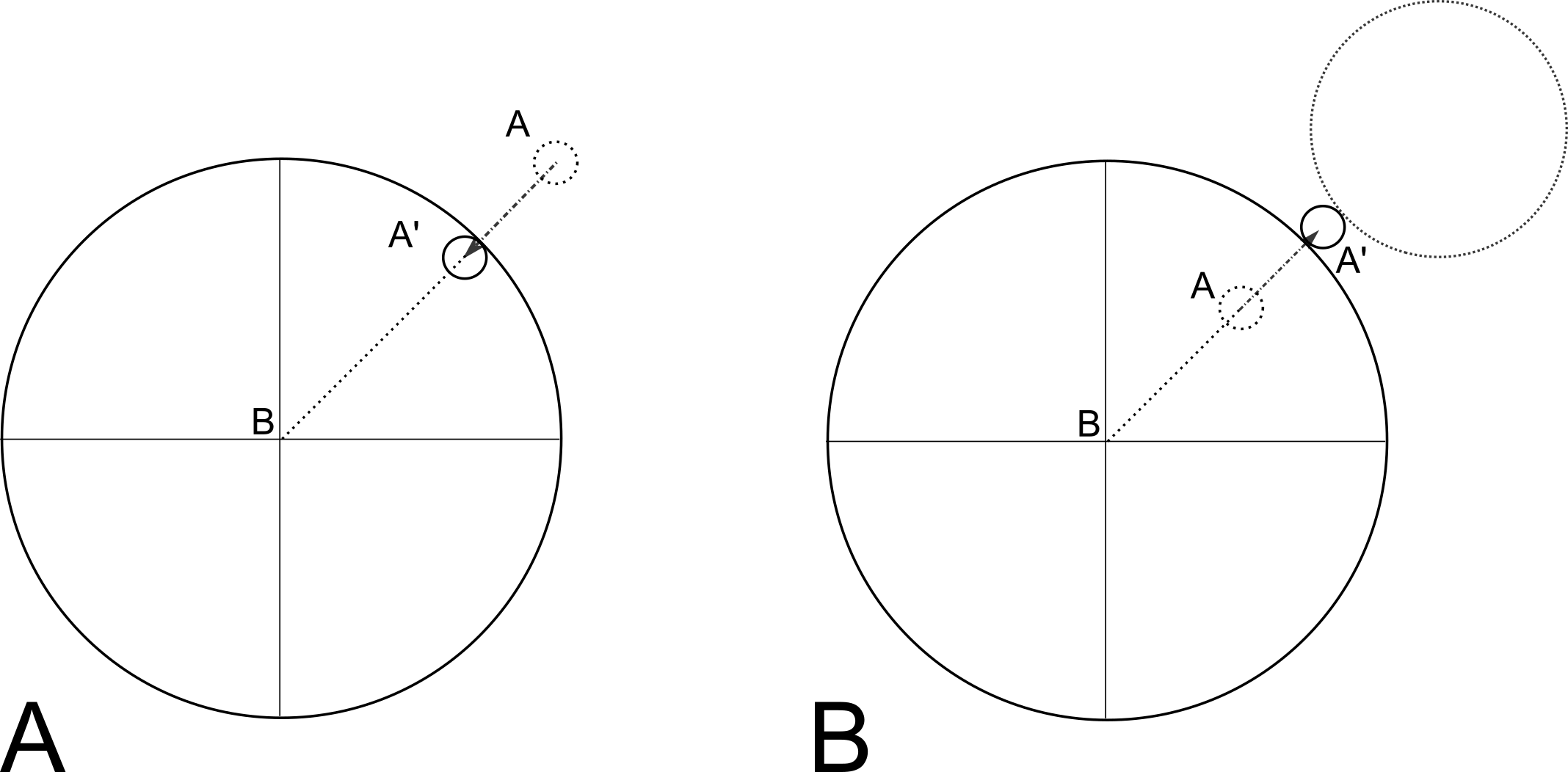

Supplement: Supplementary file 1 — Diagram of the force-directed layout constraint algorithm. The diagram represents the basic concept about how nodes’ positions are redefined by our constraining algorithm during the force-direct layout iterations. It shows how a node is moved from cytosol to the inner-border of an organelle defined in its Selected CC attribute (A) and how a node that should be in the “cytosol” is moved from an organelle to its outer-border (B). (PNG 74.0 kb) [file 12859_2017_1787_MOESM1_ESM.png]

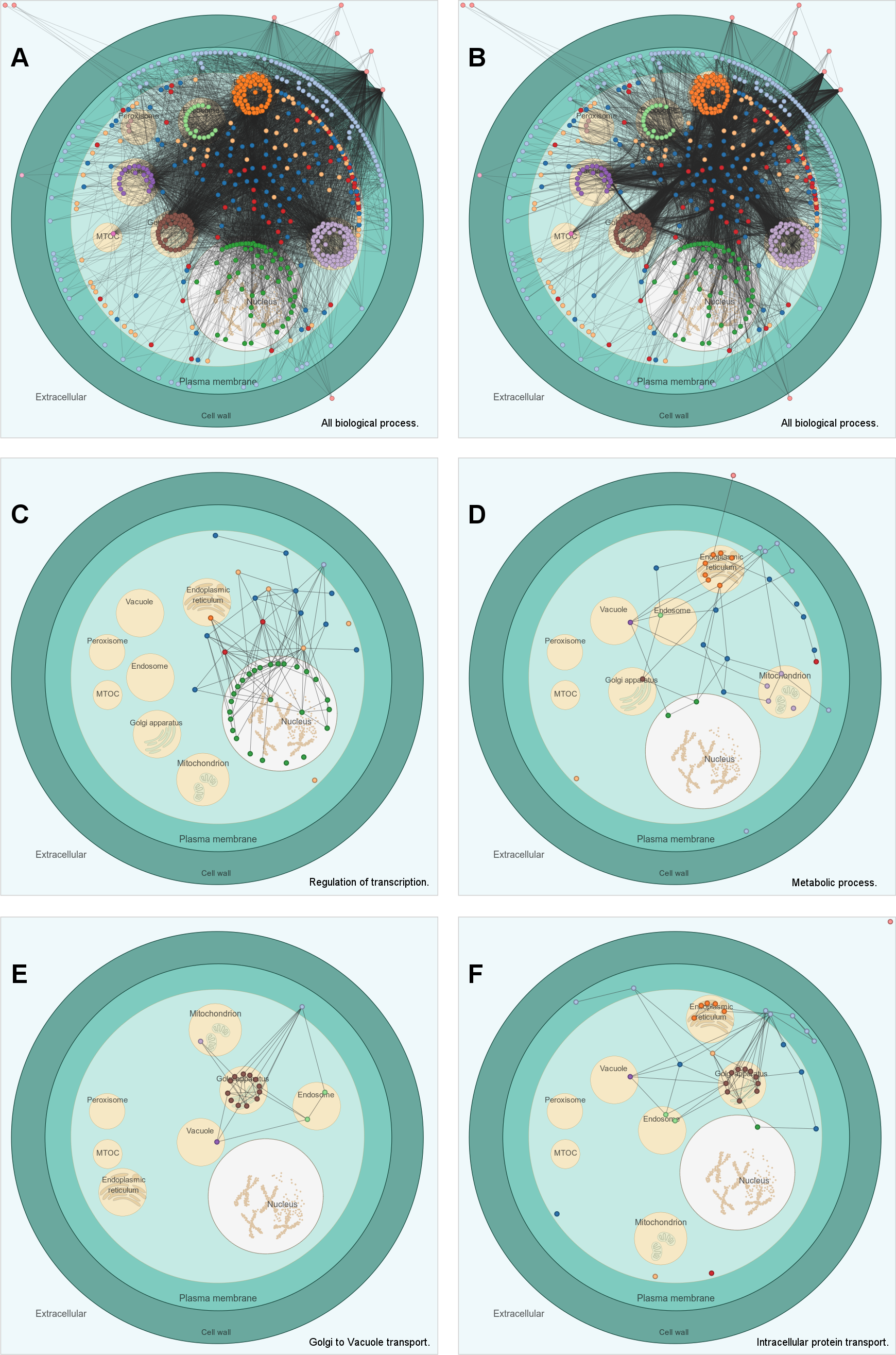

Supplement: Supplementary file 6 — Visualization of Yeast subnetworks filtered by specific biological processes. (A) and (B) represent the complete Yeast network formed by 642 nodes and 7785 edges. The network was filtered according to the following biological processes: ’regulation of transcription’ (C), ’metabolic process’ (D), ’golgi to vacuole transport’ (E), and ’intracellular protein transport’ (F). (TIF 2.59 kb) [file 12859_2017_1787_MOESM6_ESM.tif]

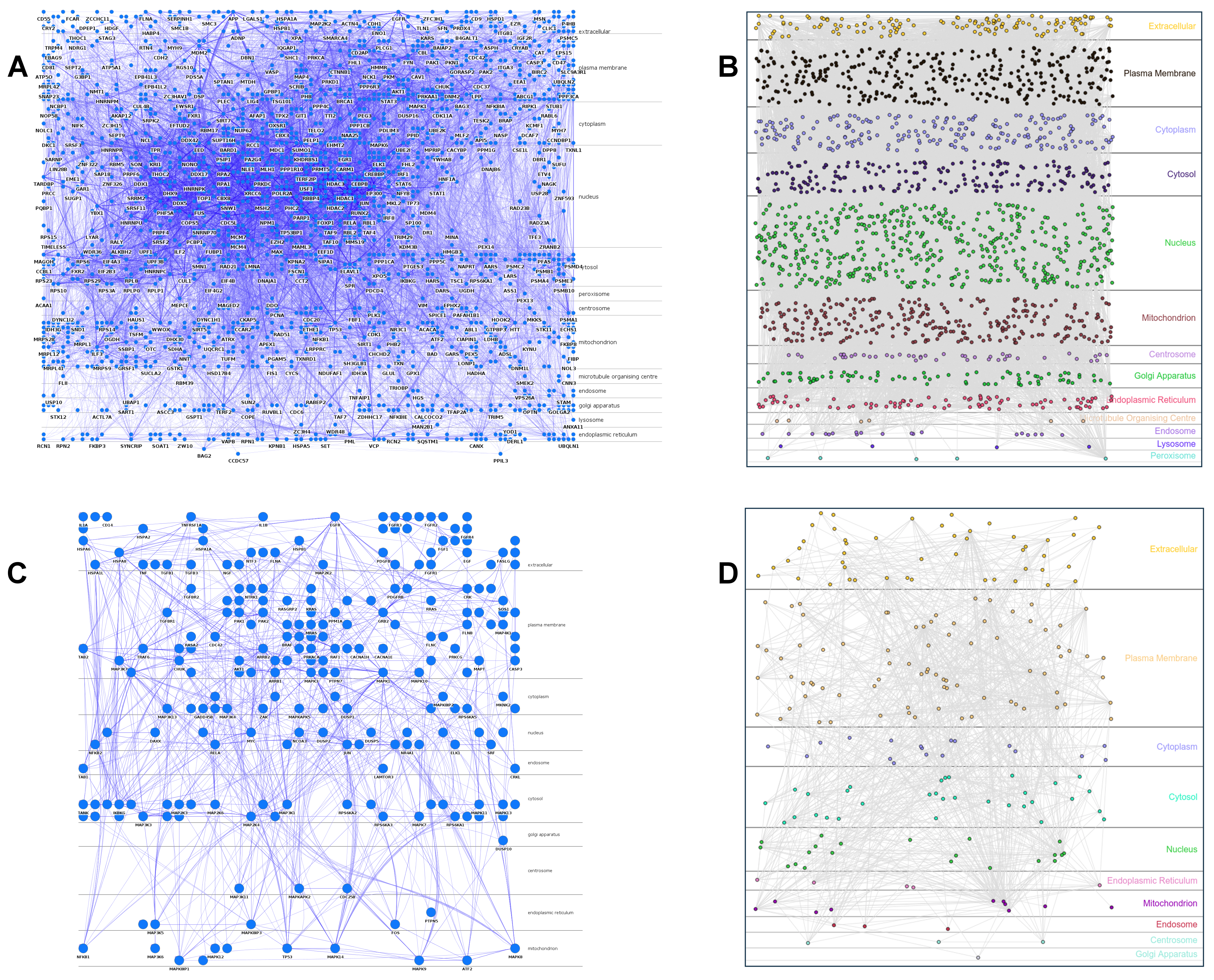

Supplement: Supplementary file 7 — Visualization of a large and of a small network on the Cerebral Cytoscape plugin (A and C) and on CerebralWeb (B and D). (A and B) Large network generated from the HPA supportive data. The drawing took approximately 3.5 min. in Cerebral (A) and 6 s. in CerebralWeb (B). (C and D) Small network generated from the human MAPK signaling pathway from KEGG database. The drawing took approximately 5 s. on Cerebral (C) and 1 s. on (D). HPA: Human Protein Atlas; MAPK: Mitogen-activated protein kinases. (TIF 14.1 kb) [file 12859_2017_1787_MOESM7_ESM.tif]

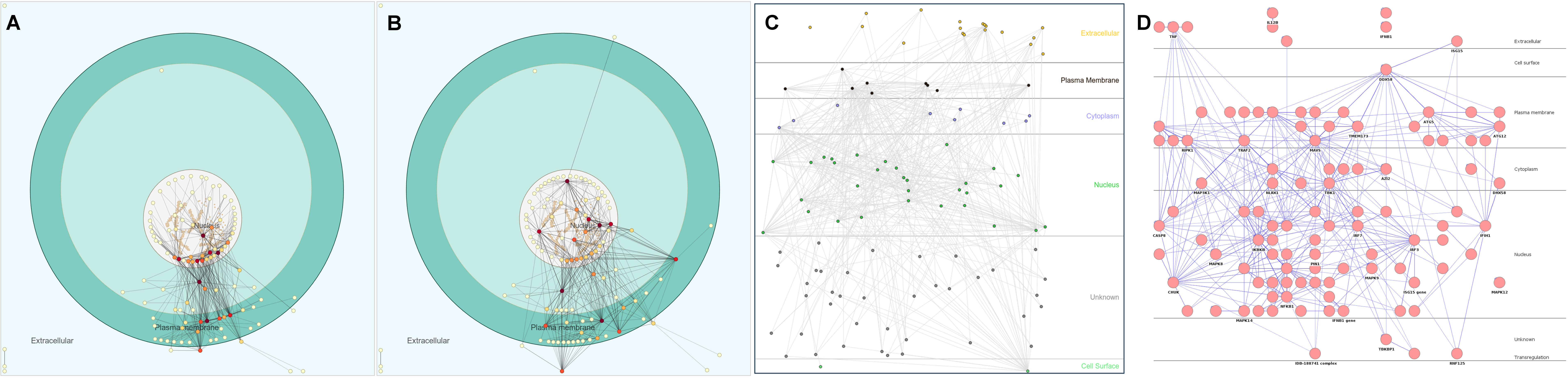

Supplement: Supplementary file 8 — Visualization of the RIG-I-like receptor signaling pathway. Visualization of the network formed by the interactions within the “RIG-I-like receptor signaling pathway (KEGG)” in Homo sapiens, downloaded from InnateDB (http://innatedb.ca/interactionSearch.do?from=pw&exPathwayXref=&pathwayFilter=5713&pathwayXrefDB=&pathwayXref=&listType=interaction&coreInteractors=true) as a XGMML file and loaded on CellNetVis, CerebralWeb and Cerebral. (A and B) Visualization of the network on CellNetVis before (A) and after (B) manually separating nodes with high degree (dark red). The same network was draw on CerebralWeb (C) and Cerebral (D) for comparison. CellNetVis was shown to be a more flexible tool through user interaction. (TIF 5.92 kb) [file 12859_2017_1787_MOESM8_ESM.tif]
